#  Green Tea *(Camellia sinensis)* Supplementation to Diabetic Rats Improves Serum and Hepatic Oxidative Stress Markers 

**Published:** 2013

**Authors:** Fatemeh Haidari, Kosar Omidian, Hossein Rafiei, Mehdi Zarei, Majid Mohamad Shahi

**Affiliations:** a *Nutrition and Metabolic Disease Research Center,, Ahvaz University of Medical Sciences, 61357-15794, Ahvaz, Iran. *; b *Diabetes Research Center, Ahvaz Jundishapur University of Medical Sciences, Ahvaz, Iran. *; c *Diabetes Research Center, Ahvaz Jundishapur University of Medical Sciences, Ahvaz-Iran. *; d *Department of Food Hygiene, Faculty of Veterinary Medicine, Shahid Chamran University of Ahvaz, Ahvaz, Iran.*; e *Hyperlipidemia Research Center, Ahvaz Jundishapur University of Medical Sciences, 61357-15794, Ahvaz, Iran.*

**Keywords:** Diabetes, Green tea, Catechins, Oxidative stress

## Abstract

Diabetes is one of the most common metabolic disorders and is interrelated to oxidative stress-induced diseases. According to the role of dietary antioxidants in control and prevention of diabetes, this study was aimed to evaluate the effect of green tea extract on serum glucose levels and serum and hepatic total antioxidant capacity (TAC) and lipid (MDA) in diabetic rats. Experimental diabetes in rats was induced by intraperitoneal injection of streptozotocin (55 mg/Kg). Alcoholic extract of green tea (100, 200 mg/Kg) was given by oral gavage to normal and diabetic rats for 4 weeks. Finally, serum glucose and serum and hepatic levels of MDA and TAC were measured and analyzed statistically. Data showed that green tea extract at dose of 200 mg/Kg significantly decreased the serum glucose levels, serum and hepatic MDA concentration and increased the total antioxidant capacity in diabetic rats (p < 0.05). Green tea supplementation also increased hepatic TAC in normal rats (p < 0.05). The antihyperglycemic and antioxidative features of green tea make it an attractive candidate for the prophylactic treatment of diabetes, although further investigation is needed to determine exact dose and duration of supplementation.

## Introduction

Diabetes mellitus that is characterized by hyperglycemia is growing at an alarming rate and the number of individuals suffering from this disease throughout the world is predicted to reach 366 million by the year 2030 (1). 

There is considerable evidence that reactive oxygen species (ROS), generated as results of hyperglycemia, lead to many of the secondary complications of diabetes and oxidative damage to peripheral tissues (2, 3). 

Several observations confirmed the antioxidative, anti-inflammatory and anti-diabetic activities of some phytochemicals such as polyphenols (4, 5). Therefore, a promising approach for hyperglycemia and its complications might be a combination therapy utilizing dietary polyphenols and hypoglycemic drugs at a suboptimal dosage to minimize any potential adverse side effects. 

Recently, green tea (*Camellia sinensis*) has received a great deal of attention especially due to its content of polyphenols. Catechins, including epigallocatechin-3-gallate (EGCG), epigallocatechin (EGC), epicatechin-3-gallate (ECG) and epicatechin (EC), are major polyphenols found in green tea and possess a wide range of health promoting effects (6).

Several animals and human studies suggested that green tea consumption is effective in lowering blood glucose in diabetes population (5, 7, 8). Furthermore, antioxidative features of green tea and its catechins have been reported in several *in-vivo *studies. Skrzydlewska *et al*. demonstrated that dietary supplementation with green tea catechins can improve total antioxidant capacity (TAC) and decrease malondialdehyde (MDA) concentration, as a biomarker of lipid peroxidation, in the rat’s liver, blood and brain (9). In another study, Quine *et al*. showed that epicatechin supplementation at dose of 15 and 30 mg/Kg in diabetic rats resulted in a significant decrease in MDA levels and increase in glutathione concentration and catalase, superoxide dismutase and glutathione peroxidase activities in the liver, kidney and heart (10).

As a result, the present study is based on the hypothesis that the bioactive compounds found in green tea (*Camellia sinensis*) have anti-diabetic and antioxidant activities. Hypoglycemic and antioxidant effects of green tea have been shown before. However, there are limited evidences about its effect on tissue biomarkers of oxidative stress and very little is known about its mechanisms. The purpose of this study was to investigate the effect of two doses of green tea extract (100, 200 mg/Kg) on serum glucose levels, as well as, serum and hepatic biomarkers of oxidative stress (MDA and TAC) in streptozotocin-induced (STZ) diabetic rats.

## Experimental

*Preparation of green tea extract*

In this study, green tea (*Camellia sinensis*) leaves were collected from North region of Iran in 2010. Dried green tea leaves were identified by a pharmacognosist in herbarium of school of pharmacy, Ahvaz Jundishapur University of Medical Sciences. Briefly, the dried green tea leaves were powdered by electrical miller. In order to prepare the extract, 150 g of green tea powder was mixed with 1000 mL of 95% ethanol (1:10 w/v) and shacked constantly for 48 h. The suspension was filtered through Whatman No. 1 filter paper and the residue was extracted again and the pooled green tea extract was vacuumed and evaporated in a rotary evaporator. The dried extracts were stored at 4°C until being used. In the present study, each 100 g of dried plant yielded about 15 g of dried extract powder (extraction efficiency = 15%.).

*Animals*

In this assay, forty-eight male albino rats of wistar strain (200-250 g), aged 6-8 weeks, were obtained from Physiology Research Center of Ahvaz Jundishapur University of Medical Sciences. The animals were housed in the steel cages in an air condition room (22 ± 3°C, 55 ± 5% humidity and a 12-h light/dark cycle) and were maintained with free access to water and ad libitum standard laboratory diet.

*Study design*

The experimental animals were divided randomly into six groups (n = 8) and received the following treatment: Group 1: Non-diabetic control rats; Group 2: Non-diabetic rats treated with 100 mg/Kg green tea extract; Group 3: Non-diabetic rats treated with 200 mg/Kg green tea extract; Group 4: Diabetic control rats; Group 5: Diabetic rats treated with 100 mg/Kg green tea extract; Group 6: Diabetic rats treated with 200 mg/Kg green tea extract. A single intraperitoneal injection of 55 mg/Kg streptozotocin (STZ) (Sigma, Aldrich, USA) dissolved in citrate buffer (0.1 M, PH: 4.6) was used for the induction of diabetes. Diabetes was confirmed through the measuring of fasting blood glucose levels 4 days after STZ injection from tail vein. Rats with fasting blood glucose ≥ 250 mg/dL with glycosuria were considered diabetic. One week after the injection of STZ, green tea extract was administered orally by gavage tube for 4 weeks. In this study, DMSO 10% was used to prepare various concentrations of green tea extract and final volume of administration was 1 mL in all groups. Animals in control groups received DMSO 10% as vehicle. During the intervention, animals were carefully monitored and weighed daily.

*Sample preparation*

At the end of the study, after an overnight fasting, animals were anesthetized by light ether and sacrificed by cervical dislocation and then, blood samples were collected directly from the heart. Serum was obtained by centrifuging the blood samples at 3000 rpm for 15 min. The livers of animals were removed, weighed and rapidly washed in cold saline (0.9%) and then placed in ice-cold isotonic potassium chloride solution (1.15% KCl w/v) containing 0.1 mM EDTA. The livers were then chopped into 4-5 volumes of 50 mM phosphate buffer (pH = 7.4) and homogenized by a homogenizer fitted with a Teflon pestle. The homogenate was then centrifuged at 3000 g for 10 min, the lipid layer was carefully removed and the resulting supernatant fraction was further centrifuged at 15,000 g for 60 min at 4°C. The supernatant was stored at - 80°C until use.

*Biochemical analysis*

Serum glucose levels were determined enzymatically using standard methods by autoanalyzer SA1000.

MDA concentration in serum and liver was assayed as a biomarker of lipid peroxidation. Briefly, 0.5 mL serum was shaken with 2.5 mL of 20% trichloroacetic acid (TCA) in a 10 mL centrifuge tube. One mL of 0.67% TBA was added to the mixture, shaken, and warmed for 60 min in a boiling water bath followed by rapid cooling. Then, it was shaken into a 4 mL of *n*-butanol layer in a separation tube and the malondialdehyde (MDA) content in the serum was determined at 532 nm by spectrophotometer against *n*-butanol.

The total antioxidant capacity of serum and liver samples were assayed by commercially available kits (Randox labs, Grumlin, UK). The assay principle was based on the ability of antioxidants to quench the absorbance of the radical cation that is formed by the reaction of a chromogen with the peroxide and H_2_O_2_ (11).

*Statistical analysis*

All data were expressed as mean ± SD. The statistical significance was evaluated by independent sample t-test and one-way analysis of variance (ANOVA) using the SPSS (version 17.0) program followed by post-hoc Tukey HSD test. Values were considered statistically significant when p < 0.05.

## Results

Table 1 illustrates the effects of green tea extract on serum glucose levels in experimental groups. As it is obvious, the administration of 100 mg/Kg green tea extract to diabetic rats did not significantly reduce the serum levels of glucose (5%), but a profound reduction of glucose levels (39%) was observed in diabetic group treated with 200 mg green tea when compared to diabetic control group (p = 0.04). The results also showed that the oral administration of 200 mg/Kg of green tea extract in diabetic rats even reaches the serum glucose levels to the normal values and the difference between the mean of glucose concentration in this group and non-diabetic control group was not statistically significant. However, following the treatment of normal rats with two dosage of green tea extract (100 and 200 mg/Kg), the serum glucose concentration did not significantly change.

**Table 1:** Effect of green tea extract supplementation on serum glucose levels

**Groups**	**Glucose (mg/dL)**	**%Change**
Normal control	153.16 ± 12.73	-
Normal+green tea (100mg/Kg)	160.16 ± 20.98	+ 4.57%
Normal+green tea (200mg/Kg)	152.6 ± 11.64	- 0.36%
Diabetic control	299.14 ± 83.11	-
Diabetic+green tea (100 mg/Kg)	284.11 ± 89.14	- 5%
Diabetic+green tea (200 mg/Kg)	182.50 ± 41.20^*^	- 39%^*^

In diabetic control group, the hepatic levels of MDA, as a biomarker of lipid peroxidation, were statistically higher than those of normal control group (p = 0.000). However, the serum MDA concentration in diabetic control rats was not significantly different from that of normal control rats (Table 2). Only oral administration of 200 mg/Kg green tea extract to diabetic rats induced a significant reduction in serum and liver MDA concentrations after 4 weeks (p < 0.05 and p < 0.001, respectively).

**Table 2:** Effect of green tea extract supplementation on serum and hepatic MDA levels

**Groups**	**Serum MDA (μmol/L)**	**Change%**	**Hepatic MDA (nmol/mg)**	**Change%**
Normal control	1.42 ± 0.27	-	10.29 ± 1.84	-
Normal+green tea (100 mg/Kg)	1.31 ± 0.24	- 7.7%	8.84 ± 1.46	- 14%
Normal+green tea (200 mg/Kg)	1.34 ± 0.32	- 5.6%	9.49 ± 1.41	- 7.77%
Diabetic control	1.78 ± 0.25	-	18.14 ± 1.21^#^	-
Diabetic+green tea (100 mg/Kg)	1.63 ± 0.15	- 8.42%	15.23 ± 2.87	- 16%
Diabetic+green tea (200 mg/Kg)	1.52 ± 0.14^*^	- 14.6%^*^	12.38 ± 2.25^**^	- 31.7%^**^

^a^: All values are expressed as mean ± SD (n = 8). Independent sample t-test was used for statistical analysis. ^*^: Indicates p < 0.05; ^**^: Indicates p < 0.001 *vs. *diabetic control group; ^#^: Indicates p < 0.001 *vs. *normal control group

In non-diabetic groups, the effect of green tea extract on serum and liver levels of MDA was not statistically significant (p > 0.05).

In this study, there was a strong positive correlation between serum levels of glucose and MDA (r = 0.406, p = 0.01).

Table 3 gives the serum and hepatic total antioxidant capacity of experimental groups. At the end of the study, the serum and hepatic total antioxidant capacity in diabetic control rats was significantly lower than those of normal control rats (p = 0.000) and following the treatment of these animals with 200 mg/Kg green tea extract, a significant increase in serum and hepatic total antioxidant capacity was observed (p = 0.04 and p = 0.02, respectively). However, the total antioxidant capacity was not significantly changed in diabetic group supplemented with 100 mg/Kg green tea.

**Table 3:** Effect of green tea extract supplementation on serum and hepatic TAC levels

**Groups**	**Serum TAC (μmol/L)**	**Change%**	**Hepatic TAC (nmol/mg)**	**Change%**
Normal control	2.35 ± 0.25	-	0.51 ± 0.07	-
Normal+green tea (100 mg/Kg)	2.43 ± 0.30	+ 3%	0.58 ± 0.07	+ 13.7%
Normal+green tea (200 mg/Kg)	2.68 ± 0.49	+ 14%	0.78 ± 0.17^#^	+ 52.9%^#^
Diabetic control	1.51 ± 0.27^#^	-	0.24 ± 0.07^#^	-
Diabetic+green tea (100 mg/Kg)	1.60 ± 0.18	+ 5.9%	0.47 ± 0.14	+ 34%
Diabetic+green tea (200 mg/Kg)	2.00 ± 0.15^*^	+ 32.4%^*^	0.55 ± 0.54^*^	+ 61.7%^*^

a: All values are expressed as mean ± SD (n = 8). Independent-sample t-test was used for statistical analysis. *indicates p < 0.05 and ** indicates p < 0.001 vs. diabetic control group; and # indicates p < 0.001 vs. normal control group

A volume of 200 mg/Kg green tea extract supplementation was also able to significantly increase the hepatic total antioxidant capacity in non-diabetic rats (p = 0.006).

A significant inverse correlation is also shown between the total antioxidant capacity and glucose concentration in this study (r = -0.449, p = 0.04).

## Discussion

The current study was conducted to determine whether feeding 2 doses of green tea extract (100, 200 mg/Kg) have beneficial effect on serum glucose levels and serum and hepatic oxidative stress biomarkers (TAC and MDA) in STZ-induced diabetic rats.

Our findings clearly showed that the oral administration of green tea extract at dose of 200 mg/Kg improved the glycemic control and even reached the serum glucose levels to the normal values. The results are in agreement with other studies (7, 8). Wolfram *et al*. suggested that EGCG (epigallocatechin gallate), one of the catechins in green tea, enhances the oral glucose tolerance in severely diabetic db/db mice (8). A study by Ramadan *et al*. reported that green tea aqueous extract significantly alleviated hyperglycemia (resulting from type 1 and 2 diabetes) induced by alloxan or cholesterol-rich diet in rats (12). Babu *et al*. showed that the increase in insulin-stimulated glucose uptake, inhibition of the intestinal GLUT system and decrease in expression of genes that control gluconeogenesis are the possible mechanisms proposed for the anti-hyperglycemic effect of green tea (13). However, Wu *et al*. indicated that the supplementation of green tea catechins does not change the blood glucose concentration in normal rats, which is consistent with our finding in normal group (14). This property of green tea (*Camellia sinensis*) and its major polyphenol constituents (catechins) in normal state could be considered as an advantage for this medicinal plant. Although the elevated levels of glucose in the circulation could give rise to diabetes and possibly other metabolic disorders (15), the excessive lowering of the glucose level in the circulation, hypoglycemia, might also contribute to several adverse effects (16).

In current study, we also found that the higher concentration of glucose was interrelated to the higher lipid peroxidation and the lower total antioxidant capacity. This result confirmed a direct relationship between diabetes and oxidative stress condition. Glucose oxidation, protein glycation, formation of advanced glycation end-products and the polyol pathways are some of the major mechanisms involved in elevated oxidative stress biomarkers in diabetes (17).

Numerous studies have demonstrated that green tea and its constituent phytochemicals (catechins) possess antioxidative properties as they improve total antioxidant capacity, suppress destructive oxygen free radicals and prevent oxidative stress damage (17-20). Coimbra *et al*. demonstrated that green tea consumption significantly increases the plasma total antioxidant capacity and reduces the lipid peroxidation products, malondialdehyde (MDA) and malondialdehyde+4-hydroxy-2(E)-nonenal (MDA+4-HNE), after 4 weeks in healthy human (19). Ohmori *et al*. reported that drinking of green tea for 2 weeks decreases serum malondialdehyde-modified LDL (MDA-LDL) concentrations in healthy male non-smokers (20). Furthermore, Babu *et al*. found that green tea extract lowers the lipid peroxidase activity in heart and aorta of diabetic rats (13). There is no evidence about the effect of green tea on hepatic biomarkers of oxidative stress until now, and the present study is the first one in this regards.

In this investigation, we observed a significant increase in serum and hepatic total antioxidant capacity and decrease in MDA concentration, following the treatment of diabetic rats with 200 mg/Kg green tea extract. It is important to note that the green tea exert mostly its antioxidant effects in diabetic rats rather than normal rats. In this study, lower dose of green tea extract (100 mg/Kg) could not significantly compensate the abased total antioxidant capacity or the elevated level of MDA concentration in diabetic rats. However, in current assay, we used only two doses of green tea extract, so further studies are required to distinct the effective dose of it.

In conclusion, the administration of 200 mg/Kg green tea extract has antihyperglycemic and antioxidative properties, so, green tea can be effective in preventing diabetes complications. However, despite evidences representing promising effects of green tea in rodents, human studies are deficient and inconsistent. Therefore, this plant should be considered as an excellent candidate for future human studies on diabetes.
